# Case Report WIN-MTB-2023001 WIN International Molecular Tumor Board A 62-year-old male with metastatic colorectal cancer with 5 prior lines of treatment

**DOI:** 10.18632/oncotarget.28744

**Published:** 2025-06-17

**Authors:** Alberto Hernando-Calvo, Razelle Kurzrock, Nadia Saoudi Gonzalez, Shai Magidi, Catherine Bresson, Fanny Wunder, Giulia Pretelli, Agatha Martin Casado, Wafik S. El-Deiry

**Affiliations:** ^1^Vall d’Hebron University Hospital, Barcelona, Spain; ^2^Vall d’Hebron Institute of Oncology, Barcelona, Spain; ^3^Worldwide Innovative Network (WIN) Association - WIN Consortium, Chevilly-Larue 94550, France; ^4^Medical College of Wisconsin, Milwaukee, WI 53226, USA; ^5^IFOM ETS – The AIRC Institute of Molecular Oncology, Milan, Italy; ^6^Legorreta Cancer Center at Brown University, Providence, RI 02903, USA; ^*^These authors contributed equally to this work

**Keywords:** cancer, precision oncology, molecular tumor board, colorectal carcinoma, cancer management

## Abstract

Heavily pretreated metastatic colorectal cancer (mCRC) poses significant therapeutic challenges. Advances in molecular profiling enables personalized strategies. We present a 62-year-old male with mCRC harboring *BRAF*, *MET*, *APC*, *TP53* and *NRAS* alterations, following FOLFOX and FOLFIRI, dabrafenib plus panitumumab, and a BRAF inhibitor clinical trial, each leading to initial responses followed by disease progression.

WIN Consortium International Molecular Tumor Board (MTB), included experts from institutions across 13 countries. Enrollment in suitable clinical trials was explored but limited by availability. Personalized combinations suggested included amivantamab-vmjw (anti-MET/EGFR antibody) (one-third standard dose) (for MET amplification and due to prior response to anti-EGFR antibody), trametinib, 1 mg po daily (MEK inhibitor for *BRAF V600E* mutation), and regorafenib (may have WNT inhibitor activity relevant to *APC* mutation; VEGFR activity relevant since *TP53* alterations upregulate VEGF/VEGFR axis) starting at 40 mg po daily three weeks on, one week off. Another option was trametinib at 1 mg daily, cetuximab (EGFR antibody), 250 mg/m² IV every two-weeks, and cabozantinib (MET and VEGFR inhibitor), 40 mg po daily. FOLFOXFIRI combined with bevacizumab, or liver-directed therapies for hepatic metastases, or regorafenib with 5FU, or crizotinib (MET inhibitor) combined with regorafenib or dabrafenib, was also suggested.

This case emphasizes the critical role of comprehensive molecular profiling and personalized therapeutic approaches in managing complex mCRC. The WIN International MTB aims to provide treatment and biomarker analysis discussion with the ultimate goal of optimizing treatment efficacy by targeting specific molecular alterations, though final treatment decisions remain at the discretion of the treating physician.

## INTRODUCTION

Colorectal cancer (CRC) ranks among the most prevalent and lethal cancers globally [1]. The metastatic form of CRC presents significant challenges due to its complexity and the variability in treatment responses [2]. Advances in molecular genetics have markedly improved cancer management, facilitating more personalized treatment strategies based on individual tumor molecular profiles [3, 4].

Here, we present the case of a 62-years-old male patient with metastatic CRC (mCRC) who underwent a comprehensive multimodal treatment regimen. This patient’s tumor genomic analysis revealed aberrations in *BRAF*, *MET*, *APC*, *TP53* and *NRAS*.

*BRAF V600E* mutations in mCRC represent a distinct subset with unique prognostic and therapeutic implications [5]. This mutation is a class I mutation that activates the MAPK pathway, leading to uncontrolled cell proliferation and tumor growth [6, 7]. Specifically, the *BRAF V600E* mutation is associated with a more aggressive disease course, poorer overall survival, and a higher likelihood of resistance to standard therapies [8]. Multiple studies have also suggested that the *BRAF V600E* mutation serves as a predictor of reduced efficacy of EGFR monoclonal antibodies, such as cetuximab and panitumumab, either as monotherapy or in combination with chemotherapy [9]. The *MET* receptor tyrosine kinase, when mutated or amplified, activates the MET signaling pathway, promoting cellular proliferation and metastasis [10]. In mCRC, *MET* mutations and alterations, though less common than other mutations, can lead to resistance to standard treatments and contribute to more aggressive tumor behavior, significantly impacting disease progression and therapeutic response [11–13]. *APC* mutations are highly prevalent in mCRC, occurring in approximately 80% of cases [14]. These mutations are critical drivers of tumor development and progression, primarily through their impact on the WNT signaling pathway. The APC protein normally functions as a negative regulator of the WNT signaling pathway, which controls cell proliferation and differentiation [15]. When *APC* is mutated, it results in uncontrolled activation of WNT signaling, leading to increased cell growth and tumorigenesis [16]. *TP53* is a pivotal tumor suppressor gene frequently mutated in CRC [17]. Mutations in *TP53* often result in the loss of its tumor-suppressive functions, leading to increased genomic instability and resistance to apoptosis, which promotes tumor growth and metastasis [17–19]. The presence of *TP53* mutations is generally associated with more aggressive tumor characteristics and may impact the effectiveness of certain therapies [20, 21]. *NRAS*, a member of the RAS family of oncogenes, plays a crucial role in regulating cell signaling pathways involved in growth and survival. *NRAS* mutations can lead to continuous activation of the MAPK pathway, which promotes cell proliferation and tumor growth [22, 23]. When mutated, *NRAS* can drive resistance to anti-EGFR therapies by bypassing the effects of these drugs [24]. While anti-EGFR treatments target the EGFR pathway to inhibit tumor growth, *NRAS* mutations can activate downstream signaling pathways independently of EGFR, allowing tumor cells to evade EGFR blockade and continue proliferating [25, 26].

**Figure 1:**
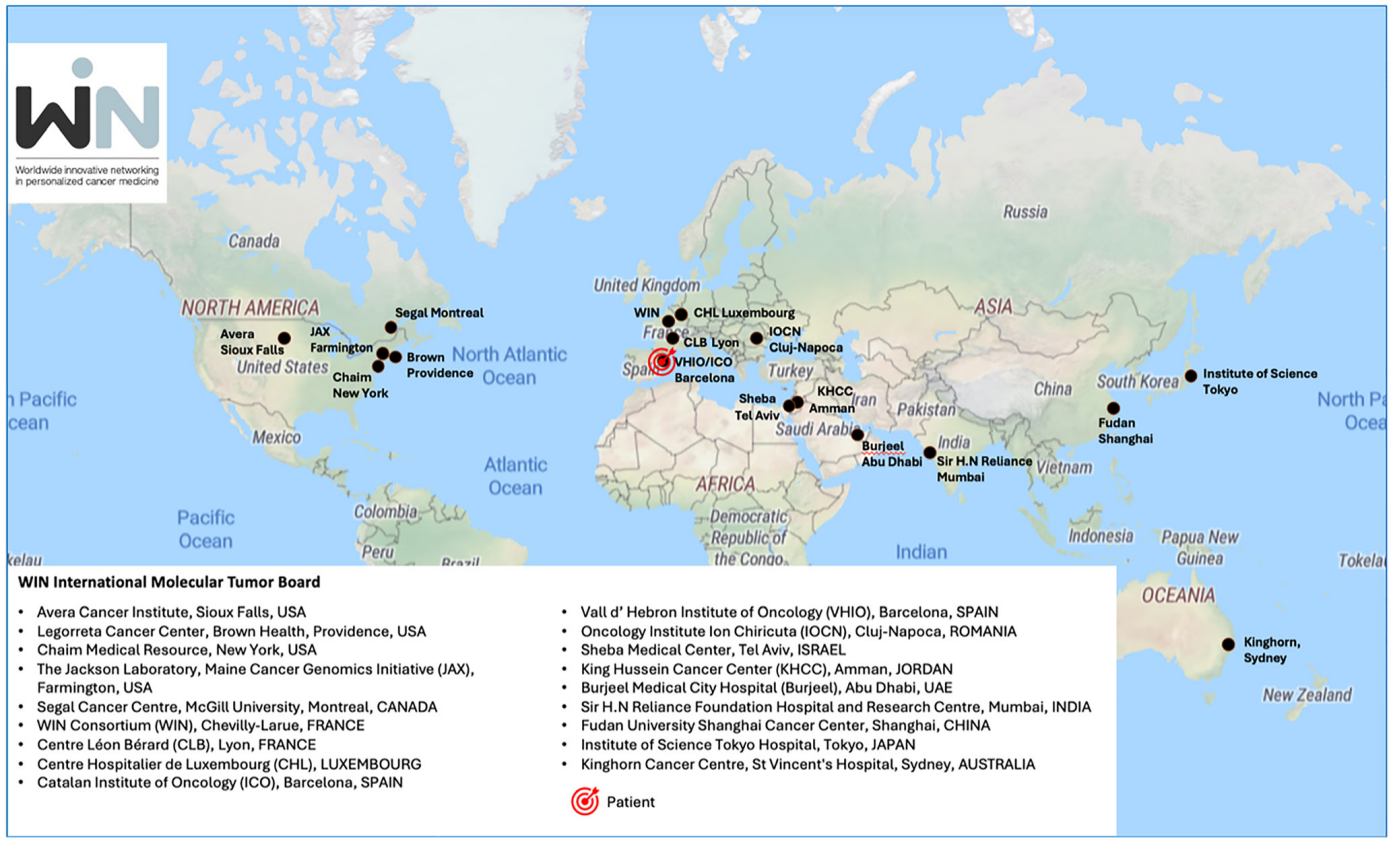
MTB international committee participants. The figure highlights 18 institutions participating in countries worldwide, with the patient discussed in the MTB originating from Spain.

The case presented here was reviewed by an international Molecular Tumor Board (MTB) convened by the WIN consortium, a French-based non-profit association comprising academic oncological centers, industry groups, research organizations, and patients advocates from multiple countries across several continents. The MTB is an international committee of expert physicians and scientists specialized in personalized cancer medicine from 18 centers across 13 countries, as shown in Figure 1. The MTB is unique in bringing together experts from multiple countries which practice in very different healthcare systems with a wide variety of drug access. The purpose of this committee is to improve the knowledge about personalized cancer therapies. As such, discussions that occurred during the MTB are for educational purposes and considered advisory only. The choice of therapy is up to the treating physician and geographic treatment availability. The board of experts evaluated the patient’s medical history and genetic profile, suggesting a variety of treatment options, including customized combination therapies, targeted treatments, standard-of-care approaches and clinical trials. The goal was to discuss treatment strategies tailored to the patient’s unique genetic molecular profile.

## CASE REPORT DESCRIPTION

### Diagnosis

The patient, a 62-year-old male, was diagnosed in May 2021 with stage IV CRC presenting with two potentially resectable liver metastases and no evidence of disease elsewhere. His medical history included high blood pressure, hyperuricemia, and kidney stones. Immunohistochemistry (IHC) performed at time of diagnosis showed that the patient was mismatch repair proficient and HER2 negative. Testing for *RAS* and *BRAF* mutations, as mandated by local clinical guidelines, identified a *BRAF V600E* mutation revealed by real-time polymerase chain reaction (RT-PCR) at the time of diagnosis.

### First line of treatment

In May 2021, shortly after his diagnosis, the patient began perioperative chemotherapy with FOLFOX (folinic acid, fluorouracil, and oxaliplatin), which resulted in a partial response. Following this response, the patient underwent surgical resection of the liver metastases. Post-surgery, the patient resumed FOLFOX chemotherapy until the primary colorectal tumor, located in the transverse colon, was also resected. The patient continued with the FOLFOX regimen for an additional two months, completing a total of 12 cycles. A subsequent CT scan confirmed the absence of recurrence. The progression-free survival (PFS) under the first line treatment of FOLFOX lasted for 10 months. However, in June 2022, a potentially resectable recurrence was identified, with two liver metastases and with no evidence of other disease sites.

### Second line of treatment

In October 2022, the patient commenced a second line of treatment of FOLFIRI (folinic acid, fluorouracil, and irinotecan hydrochloride). Given the possibility of resectable liver disease recurrence, FOLFIRI was administered without biological agents such as aflibercept or bevacizumab. Unfortunately, this treatment was discontinued after less than two months, in December 2022, due to disease progression which was confirmed by a CT scan revealing a total of 10 liver metastases. The scan also revealed a pulmonary embolism. It is important to note that the primary goal of both the first and second lines of treatment was to facilitate liver disease resection.

### Third line of treatment

In January 2023 the patient was enrolled in an early phase clinical trial involving an efflux pump inhibitor (EPI) and cancer stem-cell marker ABCG2. It is worth noting that on January 2023, the combination of encorafenib and cetuximab was not a reimbursed treatment in Spain, where the patient’s treatment center is located. Additionally, no clinical trials specifically for *BRAFV600*-mutated mCRC were available at the center or in nearby institutions. The clinical trial’s treatment was eventually discontinued due to disease progression.

At this time, results from a custom 432-gene hybrid capture-based panel (VHIO-300) [27] of a tissue sample confirmed the presence of a *BRAF V600E* mutation (Table 1), which had already been identified at the time of diagnosis. Table 1 and Supplementary Table 1 provide a list of additional detected mutations including both pathogenic/likely pathogenic variants and variant of unknown significance (VUS).

**Table 1:** Molecular alterations

Date of testing		May/2021^*^ (Diagnosis)	January/2023	May/2023	August/2023	October/2023
**Sample**		Tissue	Tissue	Blood	Blood	Blood
**Laboratory**		VHIO	VHIO	VHIO	VHIO	VHIO
**TMB (mutations/mb)** >13 is considered high		–	11.9 Low	–	–	–
**Mutations (MAF)**	APC:NM_000038.5:exon16:c.4666dup:p.T1556fs	–	–	–	13.62%	23.49%
	APC:NM_000038.6:exon16:c.4666dup:p.T1556fs	–	12%	–	–	–
	BRAF:NM_004333.4:exon15:c.1799T>A:p.V600E	^*^	14%	2.09%	41.58%	20.93%
	NRAS:NM_002524.4:exon3:c.181C>A:pQ61K	–	–	–	0.38%	0.34%
	TP53:NM_000546.5:exon10:c.1024C>T:p.R342X	–	16%	0.75%	12.43%	22.78%
**Amplifications (# of copies)**		MET	–	–	–	3.22	10.01
**Fusions**		–	–	ND	ND	–
**IHC tests**		Mismatch repair proficient, HER2 score 0	–	–	–	–
**HRD (Score)**		–	–	–	–	–
**LOH (%)**		–	–	–	–	–
**Germline testing**		–	–	–	–	–

^*^In Diagnosis (May 2021) BRAF mutation was indicated by RT-PCR. Only pathogenic alterations included; variants of unknown significance (VUS) not included. Abbreviations: IHC: immunohistochemistry; HRD: homologous recombination deficiency; LOH: loss of heterozygosity; MAF: mutant allele fraction; TMB: tumor mutations burden; ND: not detected.

Additionally, the tumor mutational burden (TMB) was measured at 11.19 mutations/megabase, which is below the high TMB cutoff of 13 mutations/megabase. This cutoff, defined through cross-validation with FoundationOne, is equivalent to 10 mutations/megabase as used typically in FoundationOne assays. Therefore, the patient did not have a high TMB.

### Fourth line of treatment

In February 2023, the patient began a compassionate use treatment with a combination of dabrafenib and panitumumab, targeting *BRAF* and *EGFR* respectively [28]. Initially, the patient showed a partial response. By the time of the documented radiological response, a liquid biopsy was performed by means of the VHIO360 ISO-certified panel using Guardant360^®^ technology [29].

ctDNA profiling showed a *BRAF* allele frequency (2.09%) (Table 1 May 2023). However, the disease eventually progressed, accompanied by an increase in BRAF allele frequency (41.58%) (Table 1 August 2023). This line of treatment lasted for five months. ctDNA molecular profiling detected *NRAS Q61K* at a low allele frequency (0.38%).

### Fifth line of treatment

In August 2023, the patient started treatment with a novel *BRAF V600* inhibitor as part of a phase I clinical trial. At the time of screening a CT scan confirmed the presence of liver metastases and, for the first time, identified lung metastases. The patient achieved stable disease as best response in September 2023 with a 13% reduction in the sum of target lesions. However, the disease eventually progressed again after two months. A new VHIO360 test after disease progression showed a *BRAF* allele frequency of 20.93% and also revealed emerging mutations and amplifications (Table 1 October 2023). Also, the number of copies of MET increased to 10.1, up from 3.2 in the previous test.

**Figure 2:**
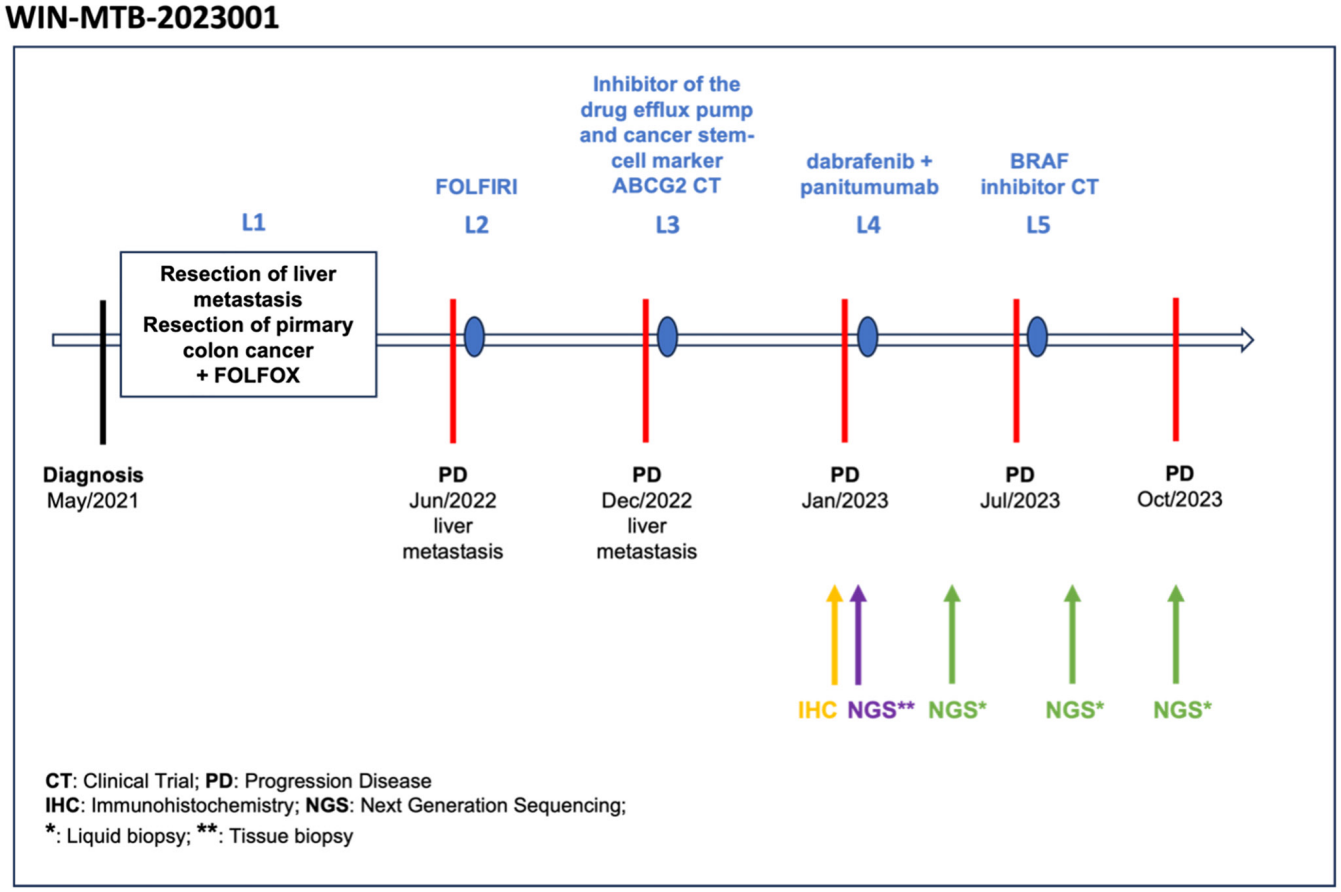
Treatment history timeline. The figure outlines the patient’s multimodal treatment timeline, including therapies, disease progression, and the timing of NGS and IHC sample analyses.

Throughout his treatment as shown in Figure 2, the patient continued to maintain an ECOG performance status of 0, remaining asymptomatic and physically active. At the time of the MTB, no new treatment had been administered while exploring further therapeutic options after MTB case discussion.

## DISCUSSION

The case presented here is clinically unique due to the complexity of the tumor’s genetic profile. At disease progression, repeat testing with both circulating tumor DNA (ctDNA) and tissue next-generation sequencing (NGS) proves valuable in identifying new genomic alterations and tracking changes in variant allele frequency (VAF). These tools not only detect emerging resistance mutations but also provide insights into tumor evolution, guiding more informed treatment decisions. While ctDNA offers a non-invasive approach to monitor tumor dynamics in real time, tissue NGS delivers a more comprehensive view of the genomic landscape. When used together, they enhance the detection of actionable targets and support a more personalized approach to cancer therapy [30–33].

The multidisciplinary discussion by the experts of the MTB aimed to discuss tailored therapeutic approaches based on the patient’s specific molecular alterations while considering its previous multimodal treatments. The discussion presents a list of therapeutic strategies prioritized by their expected efficacy. While standard-of-care options are considered, they may not appear first if they are not deemed the most suitable match for the patient. Matched therapy, where treatments are tailored to specific molecular alterations in a patient’s tumor, has shown significant benefits in improving clinical outcomes compared to non-matched therapies, especially in heavily pretreated cancer patients. Two landmark studies, I-PREDICT and WINTHER, provide key evidence for this approach [34, 35]. In the prospective I-PREDICT trial, patients received therapies matched to their tumor’s molecular alterations, resulting in improved clinical outcomes, including higher response rates and longer survival. Notably, patients who received a tailored therapy with a higher degree of matching, experienced significantly better progression-free survival than those with fewer matched alterations. The WINTHER trial, which utilized both genomic and transcriptomics data to guide personalized therapies, further demonstrated the feasibility of implementing matched therapy and improving clinical outcomes in patients with advanced cancers.

Also, it is important to mention that as patients with mCRC progress through multiple lines of therapy, the probability of achieving meaningful tumor responses declines significantly. Numerous studies have shown that in heavily pretreated patients - such as the patient described in this case – response rates tend to be low, and OS remains poor. For instance, the pivotal RECOURSE trial [36], a Phase III study evaluating TAS-102 (trifluridine/tipiracil) in mCRC patients previously treated with at least two chemotherapy regimens, demonstrated a median OS of 7.1 months with TAS-102 versus 5.3 months with placebo. The median progression-free survival (PFS) was 2.0 months vs. 1.7 months, respectively, and the disease control rate reached 44% in the treatment arm. Similarly, the CORRECT trial [37], a phase III study assessing regorafenib in mCRC patients who had progressed on standard therapies, reported median OS of 6.4 months with regorafenib compared to 5.0 months with placebo, and a median PFS of 1.9 vs. 1.7 months, respectively. More recently, the CAROSELL trial [38], a phase II study evaluating the combination of zabadinostat and nivolumab in patients with MSS mCRC after at least two prior lines of therapy, showed a median OS of 7 months. Collectively, these findings highlight the critical need for novel, more effective treatment strategies for patients with refractory mCRC—especially in the context of later-line therapies where clinical benefit remains modest.

Prior to inclusion in the BRAF inhibitor phase 1 clinical trial, the patient had two *MET* mutations classified as VUS. The most recent liquid biopsy test at the time of the MTB discussion had identified three *MET* mutations still classified as VUS. These *MET* mutations, along with the increase in the number of copies, might have contributed to the resistance mechanism observed in the patient.

Additionally, an *NRAS* variant was detected at a very low allele fraction, raising the possibility of it being a clonal hematopoiesis of indeterminate potential (CHIP) rather than a true tumor mutation. Since the tests were performed on blood samples (liquid biopsies), without tissue confirmation, the *NRAS Q61K* mutation could either have represented a minor clone or a CHIP alteration. A tissue biopsy, if feasible, could provide crucial missing information, and particularly confirm whether the NRAS mutation is genuine.

The MTB experts discussed the possibility of enrolling the patient in a clinical trial, but no suitable trials were currently available at the treating cancer center. A clinical study involving an anti-carcinoembryonic antigen (anti-CEA) antibody-drug conjugate (ADC) was considered, but no slots were available at the time. The MTB agreed that an ADC targeting CEA with a topotecan payload could be an interesting approach if the patient is a CEA producer. Although this represents a different direction, it could be highly beneficial if the patient could participate in such a study.

Also, the question arose whether it should be worthwhile to include the patient in a new single-agent *BRAF* inhibitor trial, given the complex genetic landscape observed in recent tests and the patient’s previous treatments with two *BRAF* inhibitors, including a novel *BRAF* inhibitor that was unsuccessful. The consensus was that it might not be prudent to prioritize another BRAF inhibitor trial due to the poor response observed with previous *BRAF* inhibitors and the patient’s complex genetic profile.

During the MTB discussion, it was highlighted that prioritizing secondary clinical trials may present challenges, including limited available slots and patient ineligibility. Moreover, if clinical trial opportunities are considered, it is crucial to assess whether the use of local therapies such as hepatic arterial infusion (HAI) or palliative radiotherapy might affect the eligibility of the patient to future clinical trials.

Experts in the MTB are favoring a customized combination therapies approach when considering treatment options. This methodology has been in use since 2015 under the I-PREDICT protocol at the University of California, San Diego [34, 35, 39]. This approach involves combining therapies that are often used off-label and may not be accessible in some countries.

The patient presented with significant genetic alterations, including a challenging-to-target *APC* mutation, a previously targeted *BRAF* mutation with limited response, an *NRAS* mutation that could be crucial but might also be a minor clone or CHIP alteration, a notable *TP53* mutation, and *MET* amplifications. Given the patient’s treatment history with *BRAF* inhibitors, one proposed approach was to consider trametinib, a *MEK* inhibitor that could target both *BRAF* and *NRAS*. Even if the *NRAS* mutation is a CHIP alteration, trametinib could still be effective against both targets. The EGFR pathway is also of interest, as targeted with the previous combination of panitumumab and dabrafenib (*BRAF* inhibitor) that was administered to the patient (Line 4 – 5 months – PR). Another discussed option was to target the EGFR pathway and the *MET* alteration, for which amivantamab-vmjw, a bi-specific antibody for *MET* and *EGFR* approved in the U.S. [40], was suggested. However, the availability of this drug outside the U.S. is uncertain. The rationale behind this treatment strategy lies in the observation that MET amplification frequently emerges following anti-EGFR therapies, such as the panitumumab, which was used in the patient presented here. While MET amplification confers resistance to anti-EGFR therapies in CRC, this resistance can be overcome by MET kinase inhibitors, which restore sensitivity to EGFR blockade and significantly inhibit tumor growth [41]. Therefore, incorporating a MET inhibitor, such as amivantamab, which targets both EGFR and MET, was considered a potential therapeutic approach.

Regorafenib, a *VEGF* inhibitor, also has potential efficacy, particularly given its ability to down-regulate the WNT pathway activated by *APC* mutations, and its potential effectiveness in the context of the *TP53* alteration, which is associated with an upregulation of the VEGF-VEGFR pathway [42]. Based on these considerations, a customized combination therapy involving amivantamab-vmjw, trametinib, and regorafenib was suggested. While regorafenib is approved in this indication [43], the other two drugs are not, raising questions about the practicality of this combination. In some regions of the U.S., these drugs may be accessible, but this might not be the case elsewhere. The strategy for administering customized combination therapies involves starting with reduced doses of all drugs, a practice that has been shown to be safe with close monitoring [34]. Indeed, generally, combining multiple anticancer agents often leads to overlapping toxicities, which can be more severe than those observed with single agents. Consequently, it is rare to administer the full recommended doses of each agent in combination therapies to avoid supra-additive toxicities that are frequently observed otherwise [44]. For amivantamab-vmjw, a starting dose of about one-third of the standard dose was suggested due to its challenging tolerability. Trametinib could be started at 1 mg daily, about half of the standard dose, and regorafenib at a starting dose of 40 mg daily for three weeks, followed by one week off. Close monitoring of the patient throughout the treatment would be essential.

Another potential customized combination discussed was trametinib combined with cetuximab and an additional drug such as cabozantinib. Cetuximab was considered for its role in targeting the EGFR feedback loop, while cabozantinib was chosen for its dual inhibition of *MET* and *VEGFR* [45]. For this combination, it was suggested to start trametinib at 1 mg daily, cetuximab at half the usual dose (250 mg/m^2^ intravenously every two weeks in the U.S.), and cabozantinib at an initial lower dose of 40 mg daily, as the approved dose is often too high.

A different direction was to explore more standard treatment regimens. Given the patient’s good performance status and the presence of metastases beyond the liver, such treatments could be considered. Since the patient has not previously received antiangiogenic therapy, a regimen like FOLFOXFIRI combined with bevacizumab could be an option, as it is generally well-tolerated [46]. Additionally, depending on the extent of the liver disease, consulting with specialists in liver-directed therapies might be beneficial. If the metastases were confined to the liver, some clinicians might consider hepatic arterial infusion (HAI) pumps or alternating systemic chemotherapy with intrahepatic therapy [47, 48]. Although HAI therapy has become less popular in recent years, it remains effective and could be useful. However, its effectiveness is limited by the need for specialized expertise that may not be available at all treatment centers. Other liver-directed therapies, such as radiofrequency ablation and chemoembolization, could also be considered later, although some practitioners might use these methods earlier in the course of the disease. While these approaches are less relevant for tumors outside the liver, if systemic chemotherapy was being administered and there was a significant TMB in the liver with progression, HAI and other liver-directed therapies could be considered and carefully discussed with liver experts to make sure that such therapy would not be an exclusion criterion for future potential clinical trial options. Indeed, it was highlighted that many clinical trials exclude patients based on the number or type of prior therapies received [49]. This can limit access to potentially beneficial trials for patients who have undergone multiple treatments. Also, certain therapies can impair organ functions, making patients ineligible for enrolling later on trials that require specific organ function benchmarks [50]. In addition, treatments may deteriorate a patient’s performance status, disqualifying them from trials that require better ECOG [51, 52]. These challenges often require adopting strategies to mitigate these hurdles. For example, oncologists should consider potential future trials’ eligibility when determining full treatment plans for the course of the disease.

Another option discussed was to combine crizotinib, which targets both *MET* and *ALK* with other drugs, such as regorafenib or dabrafenib. The question remains whether focusing on targeting *MET* should be a priority at this stage. Regarding regorafenib, a dose of 40 mg, as previously suggested, may be effective for some patients, but in combination strategies, dose escalation to 80 mg could be explored if tolerated by the patient. The ReDOS study demonstrated that starting with lower doses and escalating if tolerated can be effective [53].

Dabrafenib, a *BRAF* inhibitor could also be considered in combination with trametinib (*MEK* inhibitor), as the combination has already been used in *BRAF*-mutated colorectal cancer patients, particularly when these drugs were first introduced [54].

In general, regorafenib as a monotherapy is not favored due to its limited duration of effectiveness. However, there is experience using regorafenib both as a monotherapy and in combination with 5-fluorouracil (5FU), although this combination has not been fully tested [55]. For patients who progress after regorafenib, adding 5FU has been shown to be an effective regimen. Therefore, regorafenib with 5FU could be also an option.

Other potential treatment directions discussed included combining FOLFOX with immunotherapy.

It is important to note that discussions held during the MTB were for educational purposes only. The ultimate decision regarding the patient’s treatment is made exclusively by the treating physician of the patient.

While the MTB is primarily for educational purposes, there is no obligation/or in some cases ability for the treating physician to follow the treatment options discussed at the MTB. However, specifically for the case presented here, follow up was available. The patient received regorafenib at from December 2023 to February 2024, with the best response being progression in the lungs and liver. Following this progression, the patient’s ECOG performance status deteriorated, and passed away in April 2024.

One limitation was that the trajectory of tumor markers was not included, as the patient received multiple treatments across multiple institutions with varying units and reference ranges, making data availability and consistent interoperations challenging. Another limitation is the lack of ctDNA tumor fraction data at different time points. In the VHIO360 liquid biopsy panel, tumor fraction information is unavailable. However, current guidelines also recommend the use of the mean or maximum VAF of detected somatic mutations at each timepoint to monitor ctDNA kinetics [56]. However, Guardant360 tests do not provide an estimate of tumor fraction. Since the VHIO360 liquid biopsy panel is based on Guardant360 technology, tumor fraction information is similarly unavailable.

In conclusion, the WIN consortium’s MTB encompassing stakeholders from all over the world discussed several treatment options and emphasized the importance of tailoring treatment strategies and using combinations to limit escape mechanism.

## SUPPLEMENTARY MATERIALS


