# Supplementary material for: Case Report WIN-MTB-2023001 WIN International Molecular Tumor Board A 62-year-old male with metastatic colorectal cancer with 5 prior lines of treatment
Source: Oncotarget. 2025 Jun 17;16:456–66. doi: 10.18632/oncotarget.28744 (PMC12175699; doi:10.18632/oncotarget.28744)
Supplement: Supplementary file 1 [file oncotarget-16-28744-s001.pdf]

Case Report WIN-MTB-2023001
WIN International Molecular Tumor Board
A 62-year-old male with metastatic colorectal cancer with 5 prior lines of treatment

SUPPLEMENTARY MATERIALS

Supplementary Table 1: Variant of unknown significance (VUS) detected from tissue biopsy in January 2023

Variant	MAF
ARID1B:NM_020732,3:exon18:c.4556G>A:p.R1519H	15%
ARID5B:NM_032199,3:exon10:c.1858G>A:p.A620T	46%
CDH1:NM_004360.5:exon16:c.2558C>T:p.S853L	50%
FGF6:NM_020996.2:exon3:c.562C>T:p.R188W	46%
FOXP1:NM_001244814.2:exon10:c.1135G>T:p.A379S	15%
IGF1:NM_001111285.3:exon2:c.107C>T:p.A36V	9%
MTOR:NM_004958.4:exon24:c.3646A>G:p.I1216V	49%
PIK3CB:NM_006219.3:exon3:c.25C>T:p.P9S	44%
RANBP2:NM_006267.5:exon20:c.5605T>C:p.S1869P	6%
RIT1:NM_006912.6:exon6:c.664_647del:p.K215fs	49%
RUNX1T1:NM_001198626.1:exon11:c.1310C>T:p.A437V	11%
RUNX1T1:NM_001198626.1:exon4:c.215C>T:p.T72I	24%

Abbreviations: MAF: Mutation Allele Frequency.
